# Time and motion study of familial bowel and breast cancer gene mutational analysis in Victoria, Australia

**DOI:** 10.1186/1897-4287-9-S1-P32

**Published:** 2011-03-10

**Authors:** David Quin, Richard Cotton, Finlay Macrae

**Affiliations:** 1 Genomic Disorders Research Centre. Howard Florey Institute, University of Melbourne, Victoria, 3052, Australia; 2 Dept of Colorectal Medicine and Genetics, The Royal Melbourne Hospital, Victoria, 3050, Australia

## Background

The time taken to search for and report on data about unclassified genetic variants (UVs) found during testing for familial bowel and breast cancer was analysed. An estimate of this activity across Victoria was calculated. Data were analysed in the context of the value provided by a common repository supporting search and reporting of genetic information.

## Method

Data was collected describing time taken to search for information on a UV, and format the findings into a report. Data collection took place over a four month period from February 2010 to May 2010. Comparison was made to the time taken to identify a previously classified variant and produce the report, and an analysis of the differences in times reported.

## Results

47 surveys were collected, representing one-third of searches performed in the data collection period. It was estimated that each laboratory encountered 150 genetic tests each year that required information on an unclassified variant to be searched for and reported on. From this the activity of searching and reporting analysed was calculated at 20 weeks’ effort across the state of Victoria each year.

Differences were observed in times reported (Table 1). This was attributable to the level of automation of the two processes:

**Table 1:** Search and Reporting Times for Unclassified Variants in Familial Bowel and Breast Cancer in Victoria, 2010: Manual Processes vs Automatic

	Manual	Automatic
**Search**	102.375	28.5
**Reporting**	60	0 (automatic)

## Conclusion

The study found that if all laboratories could benefit from the automated process, the 20 weeks’ effort to search for and report on unclassified variants found by genetic testing for familial bowel and breast cancer could be reduced to 5 weeks.

This study supports the view that the development of a common repository approach would have significant economies of scale over repositories being developed by laboratories for individual diseases, particularly as genetic testing becomes more common.

